# The British Red Cross Society's Duties in Time of Peace

**Published:** 1912-08-31

**Authors:** 


### THE ROYAL ARMY MEDICAL CORPS SECTION.

### The British Red Cross Society's Duties in Time of Peace.

In our issue of The Hospital for June 22 we offered a word of caution to the British Red Cross Society, urging it not to expend all its energies in the formation and training of Voluntary Aid Detachments, but rather to direct attention to organising itself during peace for its duties in time of war, be that abroad or at home; and in this way to educate the nation generally to a more correct knowledge and appreciation of Red Cross work. It is well known that immediately on the outbreak of war there is a keen desire on the part of everyone to take a share in the care of the sick and wounded soldiers, either by personal service or by contributions in money or material; but it has also been shown time after time that unless there is beforehand an organisation ready to take in hand these voluntary offers of personal service and to deal with all the forms of proffered aid there must ensue confusion, overlapping, and inefficiency, the result being that this valuable civilian assistance entirely fails to be, as it ought to be, a valuable adjunct to the work of the Navy and Army Medical Services. This failure to be of real help may show itself in various ways. Thus, the efforts of those anxious to give assistance may be turned into wrong channels through ignorance of the actually needed requirements, while in the case of a foreign war valuable stores of hospital necessaries and clothing for the sick and wounded may never reach those for whom they are intended owing to want of knowledge of their existence or from there being no authorised person to distribute them. Did space allow, previous campaigns could furnish illustrations of such an unfortunate state of affairs. In fact, all the lessons of the past indicate clearly and conclusively that without preconcerted and systematised arrangements made in time of peace the voluntary assistance furnished under the excitement and panic of war is bound to be unsatisfactory and to be marked by confusion.

### The Possibility of Invasion.

The people of this country have also to realise the possibility of invasion and to face the fact that demands of a very definite nature may be made upon them in these circumstances. To meet these they must also be prepared, and that they embrace a wider field than the furnishing of Voluntary Aid Detachments has been well pointed out by Sir Alfred Keogh in the address he gave at St. James's Palace in October 1909 to the Council, presidents, and county honorary secretaries of the British Red Cross Society. On that occasion he spoke as follows: "It has been recognised in every country that when an army is operating within its own territory the general population can materially assist the fighting line. Whether there be preparation in time of peace for this work or not, the population of the various villages will be required to assist in time of national danger. Only those who have studied the nature of the duties which fall upon the population in receiving, housing, and nursing the wounded will understand how great is the work that will have to be done and how essential it is that every part of that work should be carried out without confusion and with precision. In a perfectly organised system every man and woman not in the fighting force should know his or her place. There is a place for everyone, and if everyone is in his place the machinery will move without confusion. It does not require a vivid imagination to picture how great will be the sum of human suffering if there be no previous organisation and training."

These utterances of Sir Alfred Keogh emphasise in clear and impressive words the absolute need for that preconcerted and systematised preparation that we are advocating. We feel strongly that it should be at once set on foot and got into working order by the British Red Cross Society in all the different counties, so that the nation may get to know what is exactly required of it in the case of a foreign war and in time of invasion, and may also learn how to render its voluntary aid in an efficient manner and through the proper channels.

### What the Society must do.

The Society, in the first place, has very properly taken steps to obtain official recognition and to keep itself in touch with the military authorities and their requirements. In the second place, it has established a central controlling body in the shape of a Council and Executive, who have drawn up a constitution and drafted rules for the committees established in the different counties for enrolling members and associates and for collecting subscriptions and donations. There have been issued, too, by the Society several useful pamphlets which outline the nature of the assistance that the Society may be called upon to furnish in time of war; but nothing has yet been done to ensure the co-ordination of all this voluntary aid on the outbreak of hostilities or the occurrence of invasion. When it is remembered that the exact locality in which the brunt of the latter may be felt is problematical, it is essential that every county should be prepared for it and know how to act. Some of the counties may have moved in this matter, but there has been no central supervision or approval of what has been done and no definite line of action has been decided on. Were war to break out suddenly, as it may very well do, there are few counties that have committees arranged to take in hand all the different duties that may be required. We are afraid that the outbreak of hostilities would reveal the fact that all such committees would have to be formed, and it is obvious that called into existence in such circumstances they would not have the efficiency of similar bodies selected in time of peace and thus furnished with opportunities of considering their work and of framing arrangements for carrying it out. We feel satisfied that the present unpreparedness of the country for all the various branches of Red Cross work would be best overcome by the British Red Cross Society insisting on county committees organising thoroughly their individual counties. This would probably best be done by having sub-committees on invalid transport, on personnel, on buildings suitable for hospitals, on the provision of clothing, and on all other matters requiring consideration. At headquarters the Executive Committee should have similar standing committees, whose duty it would be to gather in from the counties all the information collected and classify it in such a way that it would be of some real assistance to the Navy and Army Medical Services. It must be remembered that any organisation to be successful must recognise the principle of sub-division of labour, which is best done by committees and sub-committees, this being the means by which the services of all classes of the community can be utilised. If a Society presents too much the character of a single-class institution it will lose in efficiency and will be wanting in that popularity that is so essential for success. The motto of the Red Cross Society of our allies the Japanese is "Pay your debt to your country by keeping its soldiers," and we cannot do better than aim at making our British Society a similar channel by which our countrymen and countrywomen in all ranks of life can do the same.

